# Confirmatory factor analysis of the International Pain Outcome questionnaire in surgery

**DOI:** 10.1097/PR9.0000000000000903

**Published:** 2021-03-05

**Authors:** Mauricio Polanco-García, Roser Granero, Lluís Gallart, Jaume García-Lopez, Antonio Montes

**Affiliations:** a Department of Anesthesiology, Consorci Sanitari Alt Penedès-Garraf, Barcelona, Spain; b Ciber Fisiopatologia Obesidad y Nutrición (CIBERobn), Instituto Salud Carlos III, Madrid, Spain; c Department of Psychobiology and Methodology of Health Science, Universitat Autònoma de Barcelona, Bellaterra, Spain; d Department of Anesthesiology, Hospital del Mar. IMIM (Hospital del Mar Medical Research Institute), Universitat Autònoma de Barcelona, Bellaterra, Spain

**Keywords:** Confirmatory factor analysis, Postoperative pain, PAIN OUT, International Pain Outcome questionnaire

## Abstract

**Background:**

Choosing perioperative suitable treatments requires reliable and valid outcome measurements. The International Pain Outcome (IPO) questionnaire has been widely used for quality improvement and research purposes within the PAIN-OUT network that has collected more than 550,000 data sets of postoperative patients in 200 hospitals worldwide. Our aim is to confirm psychometric properties of the Spanish version of the IPO questionnaire and its invariance by pain predictors.

**Method:**

Sample included 4014 participants within a large age range, who underwent different surgical procedures. Confirmatory factor analysis (CFA) assessed internal structure, considering invariance by sex, age, procedure, smoking, obesity, affective disorder, and chronic pain. Incremental predictive validity of factor scores on question *would have liked more pain treatment* and opioid requirement was also estimated with logistic binary regression.

**Results:**

Confirmatory factor analysis verified original structure in 3 factors measuring pain intensity and interference (F1), adverse effects (F2), and perceptions of care (F3), with good internal consistency. Multigroup CFA analysis confirmed invariance by assessed pain predictors. Good incremental predictive capacity to identify *would have liked more pain treatment* was achieved.

**Conclusion:**

Our study confirms the factor structure, supports reliability, and adds some evidence of convergent validity of the Spanish adaptation of the IPO questionnaire. The sum of scores in its main factors serves a global outcome analysis tool. Low scores in F1 and F2 with high scores in F3 would indicate optimal quality of care.

## Abstract 2

Supplemental Digital Content is Available in the Text.

The reliability and validity of International Pain Outcome questionnaire Spanish adaptation is confirmed in a large heterogeneous sample. Factor scores can be used as a global outcome analysis tool.

## 1. Introduction

Pain is one of the factors that interferes with the proper recovery of patients after surgery, yet it is one of the most challenging factors to quantify because it is a subjective multidimensional experience.^17^ One recent effort to collect and analyze postoperative related data from 200 hospitals across the world is the PAIN-OUT project, funded by the European Commission's Seventh Framework Programme (ClinicalTrials.gov: NCT02083835).^30^ It focused on 3 areas related to postoperative pain: structure, process of care, and outcomes, and aims to improve postoperative outcomes through benchmarking, quality indicators, and the best available evidence.

As an outcome measurement tool, the International Pain Outcome (IPO) questionnaire^22^ was developed in 2 phases by Rothaug et al. based on the Revised American Pain Society Patient Outcome Questionnaire.^14,22^ Using a forward–backward methodology, the English version was translated to 9 languages, including Spanish. After phase 1 analysis, they shortened the questionnaire to adapt it to the European population. From the Revised American Pain Society Patient Outcome Questionnaire, falls and sleep were combined. They eliminated the items how helpful the information was as well as the frightened and depressed items because they have a high correlation with anxious. They included the following 3 additional questions: interference with breathing, would have liked more pain treatment, and presence of previous chronic pain. Using principal component analysis, a 3-model factor structure was identified, explaining 53.8% of the total variance: pain intensity and interference, adverse effects, and perception of care.^14,22^

The general objective of this study was to evaluate the psychometric properties of the Spanish adaptation of the IPO questionnaire in a large clinical sample including patients who underwent different types of surgery. The specific objectives were as follows: (1) to obtain empirical evidence confirming the 3-factor structure for the IPO questionnaire found by Rothaug et al.^22^ (measuring pain intensity and interference, adverse effects, and perceptions of care); (2) to test invariance and differential item functioning of the 3-factor structure by the patients' sex, chronological age, surgery type, current smoking, obesity, affective disorder, and presence of chronic previous pain^27^; and (3) to estimate the incremental predictive validity of the IPO factor scores on *would have liked more pain treatment* and total morphine consumption (defined as criteria of poor pain control).

## 2. Methods

### 2.1. Participants

**Figure 1.**
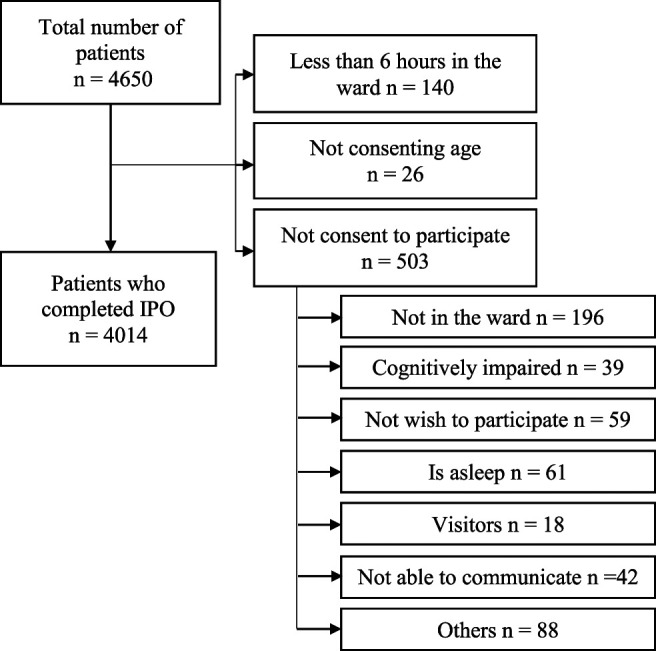
Flowchart of the study. Inclusion criteria are not mutually exclusive.

The methodology of the PAIN-OUT, a project funded by the European Commission and supported by the International Association for the Study of Pain, has been described elsewhere.^20,29^ The Spanish subsample of the European PAIN-OUT study was analyzed, which included n = 4650 patients recruited from 13 hospitals from 7 different regions of Spain. All centers were university hospitals (300–1000 beds) with an acute pain care unit. As the number of participants in the sample used for previous factorial validation studies was very low (less than 10% of the sample), we decided to maintain them in the current study to increase external validity and generalization capacity. Data were collected between February 2010 and December 2013 by trained research assistants, who followed a standard operating procedure provided by the PAIN-OUT project.^20^ Patients who accepted to participate completed the IPO questionnaire. The registered data were entered in a secured multi-institutional web-based database using a random identifier. As an inclusion criterion, it was required that patients were in their first postoperative day and in the ward for at least 6 hours. Exclusion criteria included being asleep, sedated, not in the ward at the time of data collection, and not able to communicate, including any language barrier, not able to read and understand questionnaires, and cognitive impairment (Fig. 1).

### 2.2. Instruments

The IPO questionnaire developed by Rothaug et al.^22^ and Process of Care questionnaire. This tool includes sociodemographic information, relevant pain treatment comorbid conditions, and perioperative anesthetic, surgical, and analgesic data documented in medical records. Both questionnaires are available on PAIN-OUT website (http://pain-out.med.uni-jena.de/).

### 2.3. Procedure

The study involving human subjects and the use of patient data for research purposes was approved by the Committee on Research Ethics of every participating center, and the research was conducted in accordance with the Declaration of the World Medical Association (Committee on Research Ethics Clínica Parc de Salut MAR, Reference code: No 2007/2998/I), and signed informed consent was obtained from all participants.

The measures analyzed in the study correspond to self-report measures answered by the patients in the first day after surgery at the arrival to the surgery ward. Research assistants encouraged the patients to complete the questionnaires and could read the unanswered questions to patient once, intending that the items were all answered and that no problems had occurred because of a lack of understanding.

### 2.4. Statistical analysis

**Figure 2.**
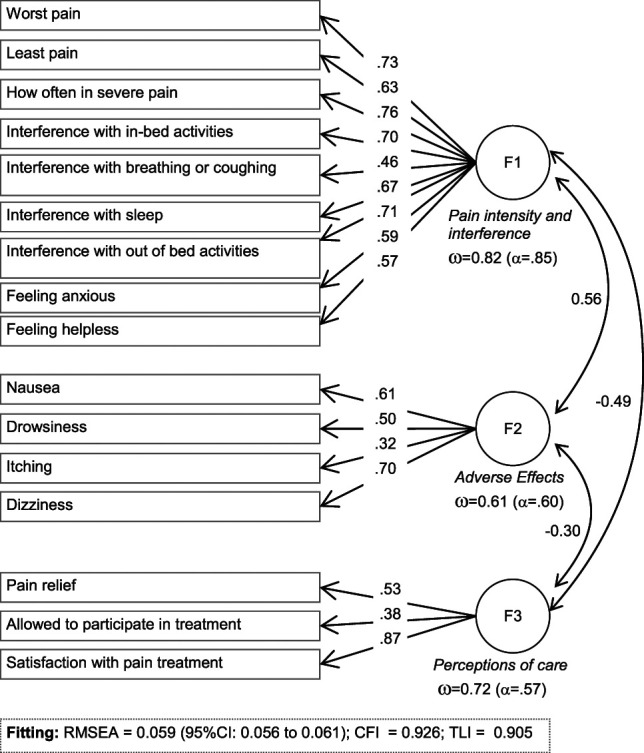
Path diagram of the CFA for the IPO questionnaire in the study (n = 4014). CFA, confirmatory factor analysis; CFI, Bentler's comparative fit index; IPO, International Pain Outcome; RMSEA, root mean square error of approximation; TLI, Tucker-Lewis index; ω, omega coefficient.

Statistical analysis was performed with Stata16 for Windows. Confirmatory factor analysis (CFA) was performed considering that the new adapted IPO questionnaire was developed after a theoretical rationale procedure based on the following steps: (1) adapting a new instrument for covering 3 specific domains (constructs/areas) related to the pain measure (intensity, adverse effects, and self-perception of care); (2) reviewing all the items initially assigned to each domain to ensure that the contents were appropriate for the target population(s); and (3) providing a meaningful distribution and order to the new version of the questionnaire. Therefore, CFA in our study was performed assuming the existence of 3 latent theoretical factors from items measures on a 0 to 10 scale: factor 1 defined by 9 items measuring pain intensity and interference(s); F2 defined by 4 items measuring pain treatment-related adverse effects; and F3 defined by 3 items assessing the patients' perception of care: allowed to participate in pain treatment decision, pain relief, and satisfaction with pain treatment, with higher scores meaning better perception of care (Fig. 2).^22^ Maximum likelihood (ML) estimator with missing values was used, and the overall goodness of fit was evaluated through the standard statistical measures^3^: the root mean square error of approximation (RMSEA), Bentler's comparative fit index (CFI), and the Tucker-Lewis index (TLI). Adequate model fit was considered for RMSEA <0.10, TLI >0.9, and CFI >0.9. The χ^2^ test was not considered as a fitting measure because of the strong dependence of this test with sample sizes (it may fail to reject inappropriate models in small sample sizes because of the lack of statistical power and it may reject appropriate models in large samples sizes because of the excess of statistical power). The internal consistency between items within each defined factor was estimated by omega coefficient (ω, this measure was considered instead of usual Cronbach's alpha because of the low number of items for the factors 2 and 3 of the IPO questionnaire) (moderate consistence was considered for consistency coefficients equal or higher than 0.60 and good consistency for values higher than 0.70). A corrected item-scale correlation was calculated for each item. Because of the strong association between statistical significance for the coefficients and sample size, a corrected item-scale correlation was considered low to poor |R| >0.10, moderate to medium for |R| >0.24, and large to high for |R| >0.37 (these thresholds corresponds to Cohen's d of 0.20, 0.50, and 0.80, respectively).^7,21^

Because of a steady association of the pain construct with variables such as sex, age (2 groups were defined based on the median in the own sample), surgery type, current smoking, obesity defined as body mass index greater or equal to 30 kg/m^2^, history of affective disorder, and presence of chronic previous pain,^23,27^ the structural configural invariance by these variables was analyzed. Given the low frequency of previous consumption of opioid analgesics, this variable was not included in the analysis. In this study, structural invariance tested factor loadings equivalence across the groups. This assumption is supported if multigroup CFA (MGCFA) analysis met the following criteria^6^: (1) the model specifying the items measuring each latent variable fits the data well; (2) all factor loadings are substantial (usually above 0.30) and statistically significant; and (3) no large modification indices exist that point to model misspecifications.

Comparison for the raw factor scores between participants' sex, groups of age, surgery type, current smoking, obesity, affective disorder, and presence of previous pain was based on analysis of variance. Effect size for the mean comparison was based on Cohen's d coefficients (low effect size was considered for |*d*| >0.20, mild to moderate for |*d*| >0.50, and high to large for |*d*| >0.80^16^).

As there are no gold standard measures of postoperative pain outcomes, the questions on the survey *would have liked more pain treatment*, either pharmacological or nonpharmacological, and highopioid requirements, defined as more than 30 mg per 24 hours morphine equivalent consumption, were used as measures of convergent validity. Opioid analgesic doses during the intraoperative and the first 24 hours after surgery, obtained from the process questionnaire, were used to calculate oral morphine equivalents consumption, based on published analgesic tables.^19^ Short-acting opioids (fentanyl and remifentanil) were part of the anesthetic protocol and not used to provide postoperative analgesia, so they were excluded. The value 30 mg per 24 hours was chosen to dichotomize the variable morphine equivalent consumption to high opioid consumption, based on previous literature.^12,20^

The incremental predictive or discriminant validity of the factor scores measured through the IPO questionnaire on the question *would have liked more pain treatment* and high opioid consumption was estimated with logistic binary regression in 2 steps or blocks: (1) the first step or block entered and fixed the patients' sex, age, surgery type, current smoking, obesity, affective disorder, and presence of previous chronic pain; and (2) the second block added the 3 raw scores in the IPO questionnaire. Goodness of fit was valued with the Hosmer–Lemeshow test (adequate fitting was considered for *P* > 0.05), whereas the incremental predictive validity of the IPO scores was estimated with the change or increase in the Nagelkerke pseudo-*R*^2^ (∆*R*^2^) comparing first and second steps or blocks of the regression and the incremental discriminant validity with the increase in the area under the ROC curve (∆AUC^2^).

## 3. Results

### 3.1. Participants

Of 4650 patients recruited, 636 did not participate in the study because of being asleep, sedated, not in the ward at the time of data collection (at least 6 hours in their first postoperative day in the ward), or not able to communicate (patient is deaf or is not able to communicate in any of the IPO available languages). Therefore, 4014 patients were analyzed. Figure 1 shows the flowchart of the study. Descriptive characteristics of the patients are shown in Table 1.

**Table 1:** Descriptive for the sample (n = 4014).

Age (years old); mean ± SD (n = 3976)	59.7	±16.3
Gender; n (%) (n = 3,990)		
Female	2346	58.8%
Male	1644	41.2%
Type of surgery; n (%) (n = 3872)		
Orthopedics	2022	52.2%
General Surgery	1713	44.2%
Others*	137	3.5%
Previous chronic pain; n (%) (n = 3912)		
Yes	2081	53.2%
No	1831	46.8%
Current smoking; n (%) (n = 3440)		
Yes	377	11.0%
No	3063	89.0%
Affective disorders; n (%) (n = 3440)		
Yes	2984	86.7%
No	456	13.3%
Obesity; n (%) (n = 3009)		
Yes	2009	66.8%
No	1000	33.2%
Preoperative opioid use; n (%) (n = 3873)		
No	3668	94.7%
Yes	205	5.3%
Liked more pain treatment; n (%) (n = 3895)		
Yes	703	18.0%
No	3192	82.0%
High opioid consumption (>30 mg/24 h oral morphine equivalents); n(%) (n = 3510)		
Yes	1013	28.8%
No	2497	71.1%
Information about treatment options; n(%) (n = 3867)		
Yes	2542	65.7%
No	1325	34.3%

* Others includes gynecology, urology, ENT, ophthalmology, cardiovascular, and thoracic surgeries.

### 3.2. Confirmatory factor analysis for the Spanish adaptation of the International Pain Outcome

Figure 2 contains the path diagram for the 3-factor model tested in the study, with the standardized coefficients obtained in the single-group CFA (whole sample, n = 4014). All the coefficients achieved high loadings with statistically significant results. Good fitting was obtained for this initial model (RMSEA = 0.059, 95% CI 0.056–0.061, CFI = 0.926, and TLI = 0.905). Internal consistency was good for the factor F1 *pain intensity and interference* (ω = 0.82), moderate for factor F2 *adverse effects* (ω = 0.61), and good for F3 *perceptions of care* (ω = 0.72). These results confirm the structure in three first-order factors for the IPO questionnaire in the whole sample (Table S1, supplementary material, contains the complete results for this CFA, as well as the frequency distribution of the raw scores for each item and for the dimension scores in the study, available at http://links.lww.com/PR9/A96).

Table S2 (supplementary material, available at http://links.lww.com/PR9/A96) contains the results to move from the single-group CFA obtained in the whole sample to the MGCFA to cross-validate the 3-factor structure across the groups defined by the participants' sex, age, surgery type, current smoking, obesity, affective disorder, and the presence of previous chronic pain. Good fitting indexes were obtained in the MGCFA models defined for assessing no differences in the structure for the IPO based on sex, age, current smoking, affective disorder, obesity, and presence of previous pain, whereas fitting was only moderate for the model measuring invariance by the surgery type. Nonsignificant results were found in the tests assessing invariance by sex (χ^2^ = 19.7, *P* = 0.104), indicating a equally statistical structure for men and women. However, invariance reported significant results in the joint test for the rest of the groups tested in the MGCFA. Examining separately the standardized coefficients in each group, significant high scores (above 0.30) were achieved for all the items (except for the item measuring itching pertaining to the factor *adverse effects*, which had a score equal to 0.265 for the group defined for other surgery types different to general and orthopedics and 0.271 for the group of men).

### 3.3. Comparison and concordance of the raw dimension scores

Table 2 includes the comparison of the mean raw factor scores measured with the IPO questionnaire in the groups considered in the study. Compared with men, women reported high mean values in the factors assessing pain intensity and interference (*P* = 0.001) and adverse effects (*P* < 0.001). These 2 factors also registered higher mean scores as lower the patients' age, whereas the factor perception of care registered lower mean for older patients compared with other 2 groups of age (*P* < 0.001). Differences for type of surgery only reported differences for the factor measuring adverse effects; orthopedic surgery reported lower mean compared with both general (*P* = 0.007) and other (*P* = 0.004) surgeries. Current smoking reported less adverse events (*P* = 0.01).

**Table 2:** Comparison for the raw factor scores between groups.

Gender →	Women (n = 2346)		Men (n = 1644)		Pairwise comparisons Women–men	
	Mean	SD	Mean	SD	*P*	|d|
F1. Pain intensity/interference	21.4	14.9	20.0	14.1	0.001*	0.09
F2. Adverse effects	7.0	7.3	4.9	5.9	<0.001*	0.31
F3. Perceptions of care	11.6	6.5	11.9	6.2	0.051	0.05

Groups of age →	16–63 y		63–109 y		Pairwise comparisons	
	G1 (n = 2092)		G2 (n = 2083)		G1–G2	
	Mean	SD	Mean	SD	*P*	|d|
F1. Pain intensity/interference	22.3	15.5	16.3	14.1	<0.001*	0.40
F2. Adverse effects	6.5	7.1	5.1	6.6	<0.001*	0.21
F3. Perceptions of care	11.4	6.8	10.1	7.5	<0.001*	0.18

Previous pain →	Without pain		With pain		Pairwise comparisons	
	n = 1831		n = 2081		Without–with	
	Mean	SD	Mean	SD	*P*	|d|
F1. Pain intensity/interference	20.6	14.3	20.9	14.9	0.755	0.02
F2. Adverse effects	6.1	6.8	6.2	6.9	0.670	0.02
F3. Perceptions of care	11.8	6.2	11.9	6.5	0.784	0.03

Surgery type →	Orthopedics		General		Others		Pairwise comparisons					
	G1 (n = 2022)		G2 (n = 1713)		G3 (n = 279)		G1–G2		G1–G3		G2–G3	
	Mean	SD	Mean	SD	Mean	SD	*P*	|d|	*P*	|d|	*P*	|d|
F1. Pain intensity/interference	20.4	14.0	21.2	15.1	21.3	15.2	0.114	0.05	0.333	0.03	0.878	0.02
F2. Adverse effects	5.8	6.8	6.4	6.9	7.1	7.2	0.007*	0.09	0.004*	0.19	0.138	0.09
F3. Perceptions of care	11.6	6.4	11.8	6.3	11.9	6.4	0.412	0.02	0.476	0.05	0.774	0.01

Current smoking →	No		Yes		Pairwise comparisons	
	n = 3063		n = 377		No–Yes	
	Mean	SD	Mean	SD	*P*	|d|
F1. Pain intensity/interference	21.0	15.0	21.1	15.1	0.569	0.01
F2. Adverse effects	6.3	6.9	5.4	6.9	0.010*	0.13
F3. Perceptions of care	11.6	6.4	10.8	7.5	0.008	0.13

History of affective disorder →	No		Yes		Pairwise comparisons	
	n = 2984		n = 456		No–Yes	
	Mean	SD	Mean	SD	*P*	|d|
F1. Pain intensity/interference	20.9	14.9	22.1	15.6	0.950	0.08
F2. Adverse effects	6.2	6.9	6.2	7.0	0.587	0.01
F3. Perceptions of care	11.6	6.5	11.2	7.1	0.106	0.06

Obesity →	No		Yes		Pairwise comparisons	
	n = 2009		n = 1000		No–Yes	
	Mean	SD	Mean	SD	*P*	|d|
F1. Pain intensity/interference	21.3	14.6	21.7	16.4	0.790	0.03
F2. Adverse effects	6.4	7.0	6.2	7.0	0.206	0.03
F3. Perceptions of care	11.5	6.3	11.8	6.5	0.890	0.05

* Bold: significant comparison (0.05 level).

†Bold: effect size into the moderate (|d| > 0.50) to high range (|d| > 0.80).

### 3.4. Incremental predictive or discriminative capacity of the International Pain Outcome

Table 3 includes the final result of the logistic regression measuring the incremental predictive validity of the IPO measures after considering the patients' sex, age, surgery type, history of affective disorders, current smoking, obesity, and presence of previous pain. For *would have liked more pain treatment* and for high opioid consumption, goodness of fit was achieved for the final model for the second step or block (*P* = 0.093 and *P* = 0.291, respectively). Increase in the pseudo-R^2^ after including and fixing the variables defined into the first step or block indicated that the specific incremental predictive capacity of IPO factor scores was around 23% (∆*R*^*2*^ = 0.227; global predictive capacity for the final model was *R*^*2*^ = 0.248) and the specific incremental discriminative capacity was also around 23% (∆AUC = 0.228; global discriminative accuracy for the final model was AUC = 0.829) in *would have liked more pain treatment*. The predictive capacity of the variables included in the model for high opioid consumption was low (*R*^*2*^ = 0.057 and AUC = 0.626) and did not increase noticeably with the inclusion of factor scores (*∆R*^*2*^ = 0.027 and ∆AUC = 0.031). Significant OR coefficients were achieved for the factors measuring pain intensity and interference (OR = 1.076, *P* < 0.001) and perception of care (OR = 0.909, *P* < 0.001) in *would have liked more pain treatment*, whereas in high opioid consumption, significant OR coefficients were achieved for pain intensity and interference (OR = 1.012, *P* < 0.001) and adverse effects (OR = 1.014, *P* < 0.001), indicating that the probability of liking for more treatment was higher for patients who perceived higher pain intensity and interference and lower perception of care, while the probability of high opioid consumption was higher for patients who perceived higher intensity and interference and higher adverse effects.

**Table 3:** Incremental predictive validity of the IPO on the criterion would have liked more pain treatment: logistic regression in 2 steps or blocks.

	B	SE	*P*	OR	95% CI (OR)		H-L	∆R^2^	∆AUC
Model 1 (would like more pain treatment)									
First step/block							0.394	0.021	0.601
Gender (0 = women; 1 = men)	0.504	0.121	<0.001*	1.656	1.306	2.099			
Age (y)	−0.007	0.004	0.052	0.993	0.986	1.000			
Previous pain (0 = no; 1 = yes)	−0.092	0.127	0.472	0.913	0.711	1.171			
Surgery orthopedics vs general	0.824	0.133	<0.001*	2.281	1.757	2.961			
Others vs general	0.310	0.268	0.247	1.364	0.806	2.307			
History of affective disorders (0 = no; 1 = yes)	0.284	0.167	0.089	1.328	0.958	1.841			
Current smoker (0 = no; 1 = yes)	0.200	0.184	0.277	1.222	0.851	1.754			
Obesity (0 = BMI <30; 1 = BMI ≥ 30)	−0.036	0.122	0.765	0.964	0.760				
Second step/block							0.093	0.227	0.228
F1. Pain intensity/interference	0.076	0.004	<0.001*	1.076	1.068	1.083			
F2. Adverse effects	0.005	0.008	0.554	1.007	0.993	1.021			
F3. Perceptions of care	−0.107	0.010	<0.001*	0.909	0.895	0.922			
Model 2 (high opioid requirements)									
First step/block							0.033	0.057	0.626
Gender (0 = women; 1 = men)	0.159	0.095	0.092	1.173	0.974	1.412			
Age (y)	−0.008	0.003	**0.008**	0.992	0.987	0.998			
Previous pain (0 = no; 1 = yes)	0.280	0.102	**0.006**	1.323	1.084	1.615			
Surgery orthopedics vs general	0.510	0.104	**<0.001**	1.665	1.358	2.041			
Others vs general	0.360	0.104	0.071	1.434	0.969	2.121			
History of affective disorders (0 = no; 1 = yes)	0.007	0.136	0.959	1.007	0.771	1.316			
Current smoker (0 = no; 1 = yes)	0.204	0.152	0.180	1.226	0.910	1.652			
Obesity (0 = BMI <30; 1 = BMI ≥ 30)	0.609	0.094	<0.001	1.838	1.530	2.208			
Second step/block							0.291	0.027	0.031
F1. Pain intensity/interference	0.012	0.003	**<0.001**	1.012	1.006	1.018			
F2. Adverse effects	0.027	0.007	**<0.001**	1.027	1.014	1.041			
F3. Perceptions of care	0.002	0.008	0.834	1.002	0.987	1.017			

H-L, Hosmer–Lemeshow test (*P*); ∆R^2^, change or increase in Nagelkerke pseudo-R; ∆AUC, change or increase in area under ROC curve.

* Bold: significant parameter (0.05 level).

IPO, International Pain Outcome.

## 4. Discussion

This study aims to test the psychometric validity of the IPO questionnaire in a large clinical Spanish sample with patients who underwent a broad range of surgical procedures and perioperative management.^20^ The main results of this study provide evidence about (1) the structure of the IPO questionnaire in 3-factors (*pain intensity and interference, adverse effects,* and *perceptions of care*); (2) the invariance of the structure by sex, age, surgery type, current smoking, history of affective disorder, obesity, and presence of previous pain; and (3) the capability of the factor scores to predict *would have liked more pain treatment*.

Similar to the initial IPO exploratory validation study,^22^ our CFA shows that interference with breathing and coughing was the item with the lowest factor loading (0.33) on F1 (pain intensity and interference). This could be related to the heterogeneity of the procedures included in the sample, considering that factor loading on this item increases in general compared with orthopedics procedures because limb procedures usually do not affect the respiratory system.^20^ The moderate adjustment of invariance by type of procedure could also be influenced by this item. For F2 (adverse effects), itch (0.32) is the factor with the lowest standardized coefficient, which may be due to the low frequency of occurrence and low intensity compared with other adverse effects.^20^ Itching was introduced in the IPO as one of the main adverse effects of intrathecal and epidural opioid treatments, but the frequency of occurrence in large sample studies has been around 6% to 18%,^11^ and although it is bothersome, its relevance in the functional status and morbidity of postoperative patients is arguable. Regarding perception of care (F3), the satisfaction item explains almost all the variability in that factor, in contrast to the original exploratory factor analysis by Rothaug,^22^ where the items loads were more balanced. Also, for F3, our study shows moderate internal consistency suggesting that pain relief, satisfaction, and participation in pain treatment decisions measure different aspects of postoperative experience in Spanish patients and should be treated separately. High participation and information about pain therapy, perceived by the patients, has been shown to be a predictor of less pain intensity, restriction with movement, and dissatisfaction in the German population.^18^ Unlike other countries, such as the United States, where both participation and satisfaction with treatment are high,^28^ Spanish patients, on average, perceive that they participate less in pain treatment, but despite this, satisfaction is comparable to other countries.^20^ This may be due to sociocultural differences, the fact that health care is public in Spain, and the degree of involvement that Spanish patients want to have in treatment decisions may differ from other countries.

The analysis presented here suggests invariance of the factor structure for sex, age, previous chronic pain, current smoking, obesity, history of affective disorders, and type of surgery, which implies that factor scores can be used to compare these groups. The lesser fit of the model by surgery type is consistent with previous literature that even lesser surgeries can result in significant pain and it it is likely that individual factors have a greater impact than nociceptiva burden of surgical procedures in the experience of postoperative pain and treatment outcomes.^12^ Raw scores show differences by sex in intensity and interference and adverse effects, but not in perception of care. The higher sensitivity to pain in women compared with men seems to occur after puberty,^4^ and it is believed to be related to increased occurrence of pain due to the menstrual cycle, that generates a larger painful memory network and higher pain sensitivity,^5^ a fact that could explain the increase in pain interference scores. Higher frequency of adverse effects could be associated to pharmacokinetic differences in the metabolism of analgesic medication, as shown by the fact that plasma morphine concentrations with the same dose are higher in women and exposes them to higher frequency of adverse effects.^10^ Interestingly, although men had lower scores in intensity of pain (F1) than women, in the logistic regression analysis, being a man is a predictor of *would have liked more pain treatment*.

When patients are grouped by age, the scores of the younger half of the sample are higher in intensity and interference, adverse effects, and perception of care. Gerbershagen et al. had already shown that young patients have greater postoperative pain regardless of the type of surgery.^13^ Although previous studies have shown that satisfaction is greater in older patients,^26^ Jaipaul and Rosenthal showed that this factor varied with health status, so that older adults (patients older than 65 years) with poor health status report less satisfaction with treatment compared with older adults in good physical condition.^15^ That could explain the higher perception of care by the younger sample in our results because our population study came mainly from tertiary care hospitals that treat older people with poor health status. Our raw scores do not differ with the presence or absence of previous chronic pain, which is in agreement with previous studies showing that only previous severe chronic pain is related with worst postoperative outcomes.^13,20^ Opposite to results in Yang metanalysis,^27^ patients with obesity and history of affective disorders had similar raw scores in the 3 factors and were not predictors of *would have liked more treatment*. As expected, current smoking had lower raw scores in adverse events, mainly by the decreased risk of nausea,^2^ but similar raw scores in pain intensity and perception of care.

*Would have liked more pain treatment* is used to validate the predictive capacity of the questionnaire, under the assumption that wanting more pain treatment is an indirect but specific measure of poor pain control. Our analysis shows the probability of liking more pain treatment increases with the intensity and interference score and decreases with the perception of care score. This agrees with Schwenkglenks et al.^24^ study results that showed that satisfaction with pain treatment was associated with 3 items: more pain relief, greater participation in the treatment of pain, and no desire to have received more treatment. As expected, intensity and interference score and adverse effects were also related to high opioid consumption.^25^

The main limitation of this study is the analysis of cross-sectional data, which did not allow to study the temporal reliability of the IPO measures, such as test–retest analysis. Other psychometric validations of the IPO, such as acceptability, responsiveness, divergent validity, and feasibility, were already proved in Rothaug study, so they were not analyzed here. The overlap of the sample with the previous validation study is a limitation, although CFAs are statistical hypothesis tests that evaluate if the data fit a hypothesized structural model (in this case a 3-factor structure model). Thus, the CFA is now sustained as the rational procedure used for the elaboration of the IPO questionnaire. Voluntary participation in the PAIN-OUT study makes the sample representative only of patients who attend public university hospitals. Characteristics of patients from other populations in Spain could modify the responses to the questionnaire and its factor structure.^18^ The IPO focus on postoperative pain, which is corroborated by its factor structure (the explained variance by intensity and interference factor in the Rothaug study is 36%). However, current trends derived from fast track and enhanced surgery protocols^1,8,9^ focus on the quality of postoperative recovery, especially the patient's early ability to move, which depends on other factors additionally to pain outcomes. Studies are needed to test the IPO validity in this new paradigm. Another limitation of the study is the absence of important variables related with the pain construct, such as sleep difficulties, degree of catastrophizing, and patient resilience as well as lack of presenting treatment data, such as type, doses, and combination of pharmacological and nonpharmacological treatment. Items factor loading depend on outcomes distribution, so pain treatments that significantly influence patient outcomes could have altered the present results.

The main strengths of our study are the large sample size, the inclusion of heterogeneous patients, and the use of MGCFA procedures to assess structure invariance by variables strongly related to pain. It is the first time that the IPO is conceptualized as a sum of scores in its main factors, which can serve as a global outcome analysis tool. Low scores in pain interferences and adverse effects with high scores in perception of care would indicate optimal quality of care.^17^

The total factor scores also allow simpler comparison between centers and procedures and could become an improvement tool in the quality of postoperative pain management.

Further studies will be needed to increase the convergent and divergent validity of the questionnaire with other measures related to postoperative pain and recovery from surgery.

## 5. Conclusion

In conclusion, the study confirms the 3-factors structure of the IPO questionnaire in the Spanish population attending public university hospitals, its invariance by sex, age, previous chronic pain, and type of surgery, and serves as a proof that the sum of the scores of the factorial structure predicts would have liked more pain treatment, a key aspect in patient satisfaction, along with the ability to participate in treatment decisions and a sense of providers caring for them.^24^

## Disclosures

The authors have no conflicts of interest to declare.

## Appendix A. Supplemental digital content

Supplemental digital content associated with this article can be found online at http://links.lww.com/PR9/A96.
